# A Systematic Review on the Therapeutic Effects of Ayahuasca

**DOI:** 10.3390/plants12132573

**Published:** 2023-07-07

**Authors:** Joana Gonçalves, Ângelo Luís, Eugenia Gallardo, Ana Paula Duarte

**Affiliations:** 1 Centro de Investigação em Ciências da Saúde (CICS-UBI), Universidade da Beira Interior, Av. Infante D. Henrique, 6200-506 Covilhã, Portugal; joanadgoncalves13@gmail.com; 2 Laboratório de Fármaco-Toxicologia, UBIMedical, Universidade da Beira Interior, Estrada Municipal 506, 6200-284 Covilhã, Portugal

**Keywords:** ayahuasca, therapeutic properties, natural treatments, systematic review

## Abstract

Traditional therapies, resorting to the use of plants, have acquired a great demand over the years, both for economic reasons and the preference for natural treatments. Some studies suggest that ayahuasca may have beneficial properties in treating some physical and psychological imbalances. Thus, we carried out a systematic review of studies published up to December 2022, where these themes were addressed. The search was carried out in the PubMed database, and only studies written in English and published in peer-reviewed journals were included. Thus, 228 publications were identified, of which 66 were included in the present study. The reviewed studies suggest that ayahuasca may have beneficial effects on various physical and psychological conditions, namely in the treatment of depression, anxiety and various diseases of the neurobiological system, as well as anti-inflammatory and antimicrobial properties, demonstrating its therapeutic potential. The number of studies that address this issue has also been growing, demonstrating interest in the search for alternative treatments. However, to the best of our knowledge, this is the first systematic review where all the findings of therapeutic effects associated with the consumption of ayahuasca are reviewed.

## 1. Introduction

Traditional therapies have been known since ancient civilizations [1]. This practice has been preserved over the centuries, with current knowledge derived from thousands of years of trial-and-error experiments by humans [2], which allowed for distinguishing and using the adequate species for the intended purposes [3], as well as isolating the first drugs used in modern medicine. The number of publications on the therapeutic potential of these plants or natural substances (e.g., psilocybin), including their use in patients nonresponsive to conventional approaches, is increasing every year. 

Ayahuasca is an ancient beverage that has been used for centuries by indigenous peoples in the northeast of the Amazon [4,5,6]. Originally, the tribes resorted to this beverage for therapeutic purposes and divine rituals [7,8,9,10]. It was also used by native healers to cure psychological disorders and stimulate creative thinking and visual creativity [11]. More recently, non-indigenous religious entities from countries such as Brazil, Peru, Colombia and Ecuador also resorted to this decoction for their rituals, namely Barquinha, União do Vegetal (UDV) and Santo Daime [4,12]. These last two have now spread to the United States, Asia, Africa and some European countries [12]. In recent decades, the popularity of ayahuasca has increased outside the Amazon region. It is often seen as a natural remedy that has been used for millennia to cure various ailments [13]. Thus, currently, this beverage, despite continuing to be used in a traditional way, is also consumed recreationally worldwide, as well as used in modern medicine [13].

The word ayahuasca comes from the language of the Andean region, from Quechua, and means “vine of the dead” or “vine of the soul” [11,12]. This word derives from the terms “aya”, meaning “soul” or “dead spirit”, and “waska” or “huasca” meaning “rope” or “vine” [6,7,12]. However, over time, other terms have also been used to refer to this drink, namely daime, hoasca, caapi, nate, natema and yajé, among others [5,6]. This term refers to a psychoactive beverage prepared from *Psychotria viridis* (*P. viridis*) leaves and *Banisteriopsis caapi* (*B. caapi*) stem scrapings, with an oily, thick appearance and a brownish color [14,15]. However, over the years, several variations in this decoction have been developed, and currently, several adulterants are known [7,9,10]. Presently, more than one hundred different plants used in the preparation of ayahuasca have been documented, namely *Nicotiana tabacum*, *Tabernaemontana* spp., *Datura suaveolens*, *Iochroma fuchsioides*, *Malouetia tamarquina*, *Brugmansia suaveolens*, *Psychotria carthagenensis*, *Brunfelsia* spp., *Juanulloa* spp. and *Peganum harmala*, among others [1,4]. Additionally, the use of synthetic analogs is also described, namely Moclobemide, Harmine freebase/HCl and Tetrahydroharmine freebase/HCl [6,8,16,17].

This psychoactive decoction owes its effects to the presence of *N*,*N*-dimethyltryptamine (DMT), from *P. viridis*, and β-carbolines (harmaline, harmine and tetrahydroharmine (THH)), from *B. caapi* [1]. DMT is a tryptamine with an agonistic function at serotonergic receptors (5-HT1A/2A/2C), which, when ingested alone, is metabolized by peripheral monoamine oxidase A (MAO-A) being inactivated [5,18]. On the other hand, when this substance is ingested together with β-carboline alkaloids, MAO-A is temporarily inhibited, and DMT can access the bloodstream and central nervous system, exerting its psychoactive effects [1,18,19,20]. The effects of DMT are further enhanced by the ability of THH to inhibit serotonin reuptake [11].

Physically, ayahuasca consumption is commonly characterized by vomiting, nausea and diarrhea [21]. Other alterations in the endocrine, cardiovascular and immune systems, as well as pupil size and body temperature, have also been verified [5,22]. On the other hand, with regard to psychological effects, users describe changes in the perception of time and space, visual and auditory changes, and also alterations at the cognitive level [1]. Religious experiences, such as connections with mythical entities or Gods, are also frequently reported [23]. Despite this, there are several studies that describe the therapeutic properties associated with the consumption of ayahuasca. Several studies point to the benefits of consuming this decoction at a psychological level, namely in cases of anxiety, depression, psychological disorders or addiction [1,13,24,25]. Other benefits, such as healing, anti-inflammatory and antimicrobial properties, have been pointed out to ayahuasca extracts [1,7].

In the last decades, the consumption of ayahuasca has undergone a great expansion all over the world, raising a lot of curiosity and interest from a therapeutic point of view [7,11]. Given the current interest in therapies of natural origin and traditional medicine, it is increasingly important to know the bioactive effects of plant species, namely those that can be applied as therapies or alternative treatments. Thus, in the present work, we systematically reviewed studies where bioactive properties associated with ayahuasca were investigated.

## 2. Results and Discussion

### 2.1. Study Selection

**Figure 1:**
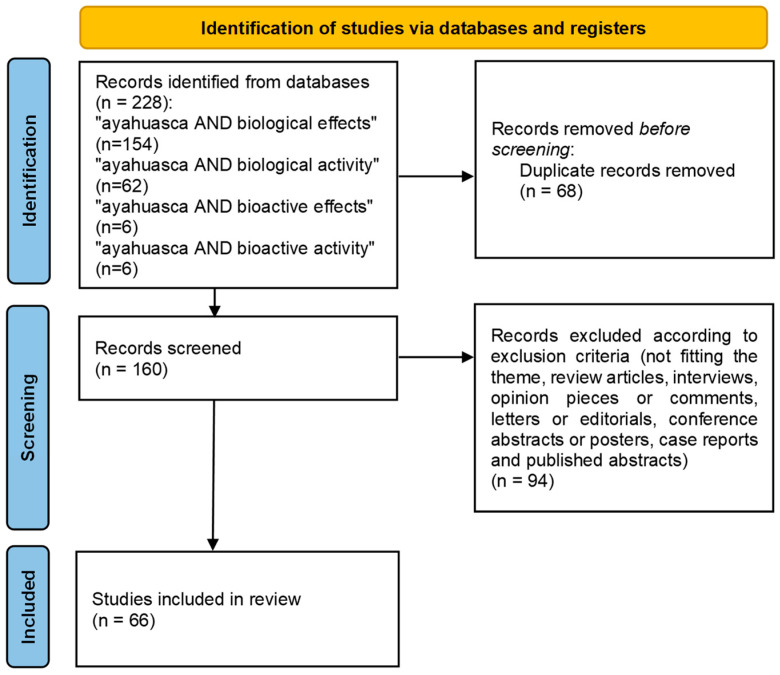
Flow-diagram illustrating the different phases of the systematic review.

Figure 1 illustrates the flow diagram with the different phases of the systematic review and respective screenings.

After performing the literature search, 228 separate references were obtained. Given the overlapping coverage between the different keywords used in the search, 68 of the publications found were duplicates. Thus, 160 publications were submitted to the first screening phase for abstract reading. After the first phase, 94 studies were excluded according to the exclusion criteria. The remaining 66 publications were submitted to the second screening phase, and after reading each publication in full, they were included in the present study.

The studies were classified according to the properties under study. The chosen studies included 27 publications on psychological/psychiatric effects, 2 studies on antimicrobial properties, 3 publications on anti-inflammatory properties, 5 studies on toxicity and toxicological effects, 9 studies on different potential therapeutic properties, 13 publications that addressed effects at the physiological level and 7 about effects at the level of metabolism. The principal characteristics of the included studies in this systematic review are summarized in Supplementary Table S1.

### 2.2. Effects Associated with Mental Health and Psychological Well-Being

Psychedelic drugs have gained interest in recent years, particularly in the treatment of psychological disorders [26]. Thus, several studies have been developed in order to find possible ways to improve anxiety and depression symptoms. A study developed by Silva et al. [27] evaluated the anxiolytic and antidepressant potential of ayahuasca in an animal model of neuroinflammation. For this, 80 male rats (90 days old) were used, divided into control groups and those with neuroinflammation induced by the application of lipopolysaccharide. Anxiety behavioral parameters were assessed by open field tests, and depressive-like behaviors were assessed by forced swimming [27]. When analyzing the results, there was a reduction in anxiety and depression behaviors, concluding that ayahuasca has an anxiolytic and antidepressant potential in this animal model of neuroinflammation [27]. Also, Correa-Netto et al. [28] investigated the effects of ayahuasca consumption on anxiety and memory in mice. For this purpose, after the last intake of ayahuasca, the animals were subjected to the open field, elevated plus maze and Morris water maze [28]. The results demonstrated that ayahuasca consumption did not affect exploration with open arms in the elevated plus maze and locomotion in the open field [28]. However, there was an increase in risk assessment behavior in the group aged between 21 and 35 days [28]. With regard to the Morris water maze, no change in spatial memory acquisition was verified [28]. However, in animals aged between 35 and 63 days, there was a reduction in the time spent in the platform quadrant [28]. Thus, the results suggested that exposure to ayahuasca in mice in childhood promoted anxiety. On the other hand, in adolescence, it promoted memory impairment; however, after reaching adulthood, these changes were not verified [28]. Studies in humans have also been described, namely the study by Sanches et al. [29], whose objective was to evaluate the potential antidepressant of ayahuasca and investigate its effects on regional cerebral blood flow. This clinical trial was carried out in a psychiatric inpatient unit, and an oral dose of ayahuasca (2.2 mL/kg) was administered to 17 patients with depression [29]. In order to evaluate the evolution of the patients, blood perfusion was performed after eight hours of administration using single photon emission tomography. The Hamilton Rating Scale for Depression, the Brief Psychiatric Rating Scale, the Montgomery–Åsberg Depression, the Young Mania Rating Scale, and Clinician-Administrated Dissociative States Scale were performed during acute effects and 1, 7, 14, and 21 days after ingestion of ayahuasca [29]. The results obtained indicated that the consumption of ayahuasca was associated with a significant decrease in scores on scales that assess depression. Additionally, increased psychoactivity and increased blood perfusion in brain regions were associated with the regulation of emotions and mood, suggesting that ayahuasca may have antidepressant properties [29]. Also, Santos et al. [30] investigated the effects of ayahuasca on panic, anxiety and hopelessness in members of a religion that uses this substance (Santo Daime). Thus, a double-blind, placebo-controlled study was carried out in which participants, who had already consumed this substance for at least ten years, were evaluated regarding their level of anxiety (state-anxiety and trait-anxiety), panic and hopelessness [30]. The participants were evaluated 1 h after consumption and the results showed that while they were under the acute effects of ayahuasca, there was no change in their state of anxiety [30]. However, states of panic and hopelessness decreased [30]. In another study developed by Mian et al. [31], the contribution of behavioral activation and mindfulness to the antidepressant effects of ayahuasca consumption was evaluated. To this end, 152 individuals were evaluated on changes in the above-mentioned parameters [31]. The analysis of results allowed us to verify that mindfulness was shown to have a strong association with the reduction in depression severity and behavioral activation a moderate association [31]. Changes in depressive symptoms were also seen on the subscales of the Five Facet Mindfulness Questionnaire and Experiences Questionnaire, and a significant improvement in Behavioral Activation for the Depression Scale-Short Form [31]. Ayahuasca consumption has been associated with curing and overcoming addictions, namely in a study carried out by Nolli et al. [32]. They used Wistar rats and evaluated whether the ingestion of ayahuasca would lead to a decrease in the ingestion of ethanol in the animals after exposure to it. Additionally, the work aimed to investigate the effects of ayahuasca on relevant neural activity in ethanol dependence [32]. Thus, the animals had access to ethanol for eight weeks, receiving ayahuasca at three different doses of ayahuasca (0.5×, 1× or 2× the dose taken during an ayahuasca ritual), naltrexone or water (control group). Another naive group only had access to water. The results revealed that the groups treated with naltrexone and ayahuasca did not lead to a decrease in ethanol intake [32]. With regard to neural activity, it was found that ethanol led to a significant decrease in cFos expression in the medial orbital cortex after treatment with naltrexone and 0.5× ayahuasca, with the latter reaching levels not significantly different from the naïve group [32]. On the other hand, ethanol led to increased cFos expression in the ventral orbital cortex in the 1× ayahuasca-treated group, in the lateral orbital cortex in the 2× ayahuasca-treated group, and in the nucleus accumbens in the naltrexone-treated group [32]. This increase was also observed in the region of the medial orbital cortex in the groups treated with naltrexone, ayahuasca 1×, ayahuasca 2× and in the control [32]. Also, Talin et al. [33] qualitatively analyzed the addiction recovery experience after ayahuasca rituals. The work was based on observation of participants in ayahuasca communities and subsequent interviews of participants with histories of substance misuse [33]. The results made it possible to verify that the effectiveness of ayahuasca in the treatment of addiction combines different dimensions (somatic, symbolic and collective), with the orientation given during the ritual being fundamental for success [33]. Thus, the authors concluded that the form and care employed play a key role in the success of addiction recovery. Additionally, inclusion in a community plays an important role in therapeutic potential [33]. Another investigation, carried out by Loizaga-Velder et al. [34], also evaluated the use of ayahuasca in the treatment of addictions. Thus, after interviewing 13 therapists who use ayahuasca for that purpose, 14 individuals who had undergone ayahuasca-assisted therapy for addictions and 2 specialist researchers were used. They found that this substance can be a tool in the treatment of substance dependence and the prevention of relapses [34]. However, the success of the treatment was conditioned by a series of variables [34]. Peláez [35] also evaluated the impact of ayahuasca consumption on personality traits of former substance users. For this, a control group and a group treated with ayahuasca were used, and it was verified that the latter presented significantly greater results in the traits of impulsivity, compassion, attachment and spiritual acceptance [35]. The same was verified in the dimensions of self-transcendence and search for novelty [35]. As far as it is known, these results may be related to the self-reflective and transcendent ritual experiences of ayahuasca, which helps in the reconstruction of personal goals, social bonds and the general projection of life [35].

Grief also seems to be improved through the ayahuasca experience. González et al. [36] developed a study where they explored the effects of ayahuasca consumption on grief. To this end, they designed a study that measured the level of grief and experiential avoidance in 30 people who participated in peer support groups compared to 30 people who took ayahuasca [36]. The results indicate that ayahuasca consumers showed benefits in some psychological and interpersonal dimensions since the level of grief was lower on the Present Feelings Scale of the Revised Texas Grief Inventory [36]. This group also described, in an open-ended question, biographical memories, emotional release and experiences of contact with the deceased [36].

Some studies describe that other important parameters related to well-being can also be influenced by the consumption of ayahuasca, namely changes in personality, concentration and tolerability, among others. Uthaug et al. [37] carried out a study whose objective was to evaluate the subacute and long-term effects of ayahuasca on well-being and cognitive thinking style, as well as to evaluate its influence on the degree of ego dissolution. The 57 participants performed the ayahuasca ritual and were evaluated the day after it, after 4 weeks and after finishing the ritual [37]. This study led to the conclusion that ayahuasca leads to improvements in affection and thinking style since the results showed that convergent thinking improved after the ceremony [37]. Likewise, life satisfaction and mindfulness increased the day after the ceremony, which was not significant after 4 weeks [37]. Levels of depression and stress were significantly lower after the ceremony and for the next 4 weeks [37]. There was also a significant correlation between changes in affection, life satisfaction and mindfulness and the level of ego dissolution achieved during the ceremony [37]. However, the same authors developed another study, this time a placebo-controlled naturalist observational study, where the influence of ayahuasca and the setting on changes in mental health was evaluated. Thus, evaluations were carried out on 30 participants before and after retreat sessions with ayahuasca [38]. The results revealed that the beneficial effects on the mental health of ayahuasca consumers may be related to non-pharmacological factors (placebo response) but also to pharmacological factors related to the use of ayahuasca [38]. Otherwise, Soler et al. [39] carried out a study where they explored the psychological mechanisms underlying the benefits of ayahuasca consumption. Thus, 25 individuals were evaluated before and after 24 h of ingestion of ayahuasca, using the Five Faces Mindfulness Questionnaire and the Experiences Questionnaire [39]. The results showed that ayahuasca ingestion led to a reduction in internal reactivity and in the processing of experience judgments and a significant increase in the ability to decentralize. These results were compatible with extensive mindfulness practice and corroborated the therapeutic potential of ayahuasca in increasing mindfulness capabilities [39]. Also, Harris et al. [40] questioned 177 individuals after using ayahuasca and found that ayahuasca users ate healthier diets, reduced alcohol intake, enjoyed improved mood and greater self-acceptance, and felt more loving and compassionate in their relationships. Seventy-four percent of respondents said they had a relationship guided and supported by the spirit of ayahuasca [40]. Barbosa et al. [41] psychologically evaluated 28 individuals one to four days before and one to two weeks after their first consumption of ayahuasca in the religious groups União do Vegetal and Santo Daime. In order to assess the state of mental health, a structured psychiatric scale was used to raise variables about attitudes toward the experience [41]. Subsequently, the second evaluation was carried out, where the state of mental health was evaluated according to the phenomenology of altered states of consciousness [41]. The results indicated positive expectations regarding the ritual, with tranquility, visual phenomena, insights, numinosity and a distressing reaction being the most notable experiences after ayahuasca consumption [41]. There was a significant decrease in psychiatric symptoms in the Santo Daime group; however, in the experiences with both religious groups, changes in serenity, vivacity/joy and assertiveness were reported [41]. In order to assess tolerability, Riba et al. [42] carried out a single-blind crossover placebo-controlled clinical trial in which they evaluated the psychological effects and tolerability of ayahuasca. Thus, three increasing doses of ayahuasca were administered to six volunteers with previous experience in the use of ayahuasca [42]. The results revealed that the effects at the psychological level start after 30 to 60 min of consumption, reaching a peak between 60 and 120 min and ceasing after 240 min [42]. Ayahuasca was found to lead to significant dose-dependent increases in five of the six subscales used [42]. It was also possible to verify that at the cardiovascular level, ayahuasca was well tolerated, and systolic blood pressure increased [42]. During the study, one of the volunteers voluntarily withdrew from the study after experiencing anxiety and intense dysphoria with transient disorientation at the intermediate dose [42].

Mindfulness and emotional stability are of great importance for psychological well-being. Domínguez-Clavé et al. [43] developed an observational study with the aim of examining the effects of ayahuasca on capabilities related to mindfulness and emotional regulation. To this end, they had 45 volunteers who participated in an ayahuasca session who were evaluated (before and after 24 h of the session) regarding emotional dysregulation (Difficulties in the Emotional Regulation Scale) and mindfulness traits (Five Facet Mindfulness Questionnaire) [43]. The results showed that participants improved in emotional non-acceptance, emotional interference and lack of control, as well as in a state of consciousness and decentration. Significant improvements were also observed in emotional interference and lack of control but not in mindfulness abilities [43]. This study suggests that ayahuasca has therapeutic potential in regulating mindfulness and emotion regulation. Also, Franquesa et al. [44] explored the relationship between ayahuasca consumption and decentralization, values and the self by conducting a comparative study between individuals with and without ayahuasca experience. The results obtained indicate that individuals with ayahuasca experience scored less in fulfillment of life, living values, self in close relationships, self in social relationships and general self [44]. However, they obtained better results for positive self and decentralization [44].

Other studies indicate that creativity and creative thinking are also influenced by ayahuasca. Kuypers et al. [26] evaluated the effects of ayahuasca on creative thinking by performing creativity tests before and during the effect of ayahuasca in 26 participants of spiritual workshops. Tests performed included the Image Concept Test, which assesses divergent and convergent thinking, and the pattern/Line Meaning Test, which assesses divergent thinking [26]. The results of the image concept test showed significant changes, inferring that ayahuasca consumption modified divergent (increased) and convergent thinking (decreased) [26]. This study led to the conclusion that ayahuasca ingestion enhances divergent creative thinking and increases psychological flexibility, which allows for facilitated psychotherapeutic interventions [26]. Also, Frecska et al. [45] studied the psychometric measures of creativity, after the disappearance of the acute effects, in ayahuasca ceremonies. Additionally, they investigated the appearance of entoptic phenomena during the expression of creativity [45]. Thus, forty participants in ayahuasca rituals were tested using Torrance Tests of Creative Thinking before and two days after the completion of two weeks of rituals [45]. The study had a control group composed of twenty-one individuals who performed the same tests [45]. When analyzing the results, it was verified that the ingestion of ayahuasca led to a significant increase in phosphenic and original responses; however, this increase already occurred in the baseline [45]. Thus, these results suggest that visual creativity and entoptic activity may increase after the acute effects of ayahuasca consumption [45].

Other studies also describe changes in spirituality and temporal reproduction. Weiss et al. [46] studied the association between the ceremonial use of ayahuasca and changes in personality traits. To this end, they resorted to the participation of individuals who attend spiritual and ayahuasca healing centers, evaluated in three moments: before, after and over three months after consuming ayahuasca [46]. The results of changes in personality and the moderation of these changes by covariates were evaluated by linear mixed models [46]. Thus, it was possible to verify that neuroticism was the major alteration; however, acute experiences and purgative experiences and moderation of personality change by baseline personality were also observed [46]. On the other hand, Campagnoli et al. [47] developed a double-blind study where they evaluated the effects of ayahuasca at two concentrations in a ritualistic context, using temporal reproduction tasks in participants with experience in ayahuasca consumption. For this, nine volunteers were asked to ingest ayahuasca in two different doses (low concentration or ritualistic concentration) and at two different moments in the ritual; they then performed the task of listening to 20 s of musical stimuli and played it immediately [47]. The results made it possible to verify that there is less temporal distortion in the participants who consumed ayahuasca [47]. Trichter et al. [48] further evaluated the influence of participation in an ayahuasca ceremony on spirituality and novice participants. Thus, participants in an ayahuasca ritual were compared with non-participants in rituals, using the Peak Experience Profile, the Spiritual Well-being Scale and the Mysticism Scale [48]. When analyzing the results, it was possible to verify that there were no significant increases in the Spiritual Well-being Scale and in the Mysticism Scale; however, it was verified that the increase in the score in the Peak Experience Profile was accompanied by higher scores in the Spiritual Well-being Scale and in the Mysticism Scale [48].

Studies to investigate the effects of ayahuasca in terms of psychological well-being are the most common, with very promising results, especially in the treatment of disorders such as depression and anxiety. However, positive results have been found in the various other purposes, which influence well-being, reported above. The summary of effects associated with mental health and psychological well-being is described in Table 1.

### 2.3. Antimicrobial Properties

The resistance of microorganisms to conventionally used drugs is a major current concern [7]. Thus, the search for alternatives, namely of natural origin, has been awakening interest among the scientific community [7]. During the literature search, two studies were found describing the antimicrobial properties of ayahuasca. In a study developed by Bussmann et al. [49], the minimum inhibitory concentration (MIC) of several plant extracts, namely *B. caapi*, and their antibacterial properties against Gram-positive and Gram-negative bacteria were evaluated. After determining the MIC values, it was verified that the ethanolic extracts of *B. caapi* showed interesting activity against *E. coli*. The same extracts were tested against S. aureus, obtaining a MIC value of 1 mg/mL [49]. In another study developed by Gonçalves et al. [7], the antimicrobial properties of nine plant extracts were evaluated against four Gram-positive bacterial strains (*S. aureus*, *B. cereus*, *L. monocytogenes* and *E. faecalis*) and four Gram-negative strains (*A. baumannii*, *P. aeruginosa*, *E. coli* and *S. typhimurium*). The results obtained during the disk diffusion test showed that six samples (*P. viridis*, *B. caapi*, *Peganum harmala* (*P. harmala*), *Mimosa hostilis* (*M. hostilis*) and a mixture of *M. hostilis* and (*P. harmala*) inhibited the bacterial growth in all strains [7]. The least susceptible strain was *E. faecalis*, with a range of inhibition diameters between 6.00 mm and 10.13 mm [7]. Regarding the resazurin microtitration method, it was verified that the samples that generally showed better MIC values were the samples of *B. caapi* and *P. harmala* [7]. With regard to anti-quorum sensing properties, only the *M. hostilis* sample did not inhibit violacein production and, consequently, the quorum sensing. Regarding the anti-biofilm activity, it was verified that in the presence of the samples of *B. caapi* and *P. harmala* the biofilm formation did not occur in the *A. baumanni* strain [7].

The results obtained in both studies (Table 2) are indicative that ayahuasca has antimicrobial effects and may be used to combat pathogenic microorganisms responsible for various infections.

### 2.4. Anti-Inflammatory Properties

Inflammation is a protective reaction developed in response to harmful stimuli; however, in the long term, it can result in the development of chronic diseases [50]. Thus, the search for compounds that inhibit the development of the inflammatory response has been increasing. Gonçalves et al. [7] evaluated the anti-inflammatory activity by the protein denaturation inhibition method in nine samples. It was verified that the samples with a lower IC_50_ value presenting, therefore, a better anti-inflammatory activity were the extracts of *P. harmala*, *M. hostilis*, a mixture of *M. hostilis* and *P. harmala*, and a commercial mixture [7]. Also, Galvão-Coelho et al. [51] performed a double-blind, placebo-controlled clinical trial of ayahuasca in 45 healthy controls and 28 patients with treatment-resistant depression. During the study, it was evaluated whether ayahuasca consumption alters inflammation biomarkers, namely C-reactive protein and interleukin 6, and the correlation with serum levels of cortisol and brain-derived neurotrophic factor was established [51]. For this purpose, blood samples were collected before and 48 h after ingestion of the substance under study in order to verify the concentration of inflammatory biomarkers. After analyzing the results, it was possible to verify that before treatment, the group of patients with depression had higher levels of C-reactive protein than the control group. A significant negative correlation was also observed between C-reactive protein and serum cortisol levels [51]. After treatment with ayahuasca, there was a significant reduction in C-reactive protein levels [51]. In another study carried out by Liu et al. [50], the anti-inflammatory effects of β-carboline alkaloids present in the plant *P. harmala*, commonly used in the preparation of ayahuasca, were investigated using a nuclear factor-kB (NF-kB) reporter assay [50]. The results indicate that harmol and harmine were able to inhibit NF-kB transactivity, with the latter inhibiting NF-kB transactivity induced by tumor necrosis factor-α and lipopolysaccharides and nuclear translocation in macrophage RAW264.7 cells mouse [50]. There was also a decrease in NF-kB mRNA and protein levels downstream of inflammatory cytokines. In the same study, a lipopolysaccharide-stimulated mouse model was also used, where harmine decreased the serum levels of tumor necrosis factor-α, interleukin-6, interleukin-1b and prevented lung inflammation [50]. Thus, these results are indicative that harmine can exert an anti-inflammatory effect by inhibiting the NF-kB signaling pathway [50]. Although, so far, few studies have evaluated the anti-inflammatory properties associated with ayahuasca, the results indicate that both the ayahuasca extracts [7,51] and some β-carbolines [50] present in it can decrease factors that contribute to inflammation. The summary of the anti-inflammatory properties is described in Table 3.

### 2.5. Other Therapeutic Effects

The therapeutic potential of ayahuasca for various conditions and diseases has been investigated over the years [32]. Most studies that report the therapeutic potential of ayahuasca are related to the effects at the neurological level. Katchborian-Neto et al. [52] developed a study whose objective was to evaluate the potential of neuroprotection conferred by ayahuasca on the cell viability of SH-SY5Y neuroblastoma in an *in vitro* model of Parkinson’s disease. Thus, initially, the cytotoxicity of the crude extracts of *B. caapi* and *P. viridis* and their hydroethanolic and alkaloid fractions was evaluated using an MTT assay (48 h and 72 h), and then the chemical composition of the samples was analyzed using ultra-performance liquid chromatography coupled with electrospray ionization and time-of-flight (UPLC-ESI-TOF) [52]. The main alkaloids were quantified by UPLC-MS/MS [52]. After analyzing the results, it was possible to verify that the samples did not present cytotoxicity *in vitro*, and in three samples, the cell viability increased after 48 h [52]. It was also verified that the crude extracts and the alkaloid fractions presented a neuroprotective effect after 72 h of exposure [52]. On the other hand, the hydroalcoholic fractions showed the same neuroprotection results, but at both times tested (48 h and 72 h) [52]. The β-carbolines and monoterpene indole alkaloids were shown to be correlated with this property, while harmine, despite showing potent neuroprotective action in 72 h, did not present a correlation with the neuroprotection profile [52]. The authors concluded that the lowest doses stimulated cell proliferation and/or had the most effective neuroprotective profile [52]. Morales-Garcia et al. [53] proceeded to develop an *in vitro* and *in vivo* study, where they evaluated the potential neurogenic effect of DMT. The results demonstrated that DMT administration promotes newly generated neurons in the granular zone through activation of the adult neurogenic niche (the subgranular zone of the dentate gyrus of the hippocampus) [53]. Additionally, it was found that the mice used in the study, treated with DMT, performed better in memory tests than the animals in the control group [53]. Thus, the study concluded that treatment with DMT leads to the proliferation of neural stem cells and the migration of neuroblasts, which promotes the generation of new neurons in the hippocampus [53]. This may be indicative of increased adult neurogenesis and improved learning tasks and spatial memory [53]. Another study developed by Samoylenko et al. [54] evaluated the potential for preventing neurological disorders, such as Parkinson’s disease, by evaluating the MAO inhibitory activities of *B. caapi* constituents. Thus, after fractionation and isolation of *B. caapi* constituents, their inhibitory activity on catechol-*O*-methyl transferase, MAO-A, MAO-B, butyrylcholinesterase and acetylcholinesterase was evaluated. Additionally, the cytotoxic and antioxidant activities of the extract and isolated compounds were also evaluated [54]. Results revealed that harmine and harmaline were able to strongly inhibit MAO-A and MAO-B *in vitro*, and (-)-epicatechin and (-)-procyanidin showed potent antioxidant and moderate MAO-B inhibitory activity [54]. The study made it possible to verify that the stem extract of *B. caapi* could help in the treatment of parkinsonism and other neurodegenerative disorders [54]. Also, in order to assess the influence of ayahuasca on Parkinson’s disease, Schwarz et al. [55] investigated the activity of *B. caapi* extract, harmine and harmaline. Thus, the effects of plant extract and compounds on rat liver MAO-A and MAO-B activity were evaluated [55]. The results showed that harmaline achieves concentration-dependent inhibition of MAO-A but demonstrated little activity on MAO-B [55]. It was also verified that *B. caapi* extract, harmine and harmaline led to a significant increase in [3H]dopamine. The results demonstrated that the extract of *B. caapi* could be promising in the treatment of Parkinson’s Disease since the discovery that harmine and harmaline stimulate the release of dopamine is a new discovery [55]. Bouso et al. [56] also investigated the neuropsychological performance in executive function and working memory after acute ingestion of ayahuasca. For this, 24 participants were evaluated in the execution of the Stroop, Sternberg and Tower of London tasks, in a usual environment, before and after ingestion of ayahuasca [56]. The results showed that there was an increase in errors in the performance of the Sternberg task; on the other hand, the reaction times in the performance of the Stroop task decreased [56]. No participant showed alterations after ingestion of ayahuasca [56]. Regarding the results of the Tower of London, there was a significant increase for experienced users in resolution and execution times and in the number of movements performed [56]. The study concluded that acute ingestion of ayahuasca decreased stimulus–response interference and impaired working memory [56]. However, it was also concluded that greater previous exposure to ayahuasca resulted in a decrease in disability, so the continued use of this substance may be related to neuromodulatory or compensatory effects on executive function [56].

Other therapeutic effects, such as skin healing and overcoming eating disorders, among others, have also been reported. Gonçalves et al. [1] evaluated the healing potential of nine plant extracts used in the preparation of ayahuasca in the NHDF cell line. Thus, a wound-healing assay was carried out, and later a Parallel artificial membrane permeability assay, in order to understand whether the compounds were absorbed by the skin fibroblasts [1]. The results allowed for verification that all samples promoted the migration of skin fibroblasts, not being absorbed through the skin [1]. Thus, the results revealed that the ayahuasca samples presented a great healing potential [1]. On the other hand, Lafrance et al. [57] developed a study where they explored the therapeutic potential of ayahuasca in eating disorders. To this end, 16 individuals with eating disorders were interviewed about their experiences with ayahuasca consumption [57]. The results of the interviews demonstrated that after the beginning of the participation in ayahuasca ceremonies, there were reductions in thoughts and symptoms related to eating disorders [57]. Other improvements in terms of reductions in anxiety, depression, self-injury, suicide and problematic substance use have also been described [57]. Participants also described that the ceremonial context enhanced the results and reduced the risks and damage that may have occurred [57]. Also, Santos et al. [58] evaluated the sensitization and acute tolerance of ayahuasca in repeated doses through a double-blind, crossover and placebo-controlled clinical trial. Thus, participants received a lactose placebo 4 h later, one dose of ayahuasca (control) or two doses of ayahuasca 4 h apart (treated) [58]. Subsequently, cardiovascular, autonomic, neurophysiological, cellular immunity and neuroendocrine measurements were performed. The results showed that, after the second dose of ayahuasca, there was a significant decrease in growth hormone and a reduction in heart rate and systolic blood pressure [58]. However, no differences were observed for autonomic, neurophysiological or immunological effects [58]. Thus, it was concluded that there was a significant tolerance to the secretion of growth hormone and less cardiovascular activation [58]. There was no sensitization or tolerance in the remaining variables [58]. Halpern et al. [59] also investigated the effects of ayahuasca on members of the Santo Daime Church in the United States of America. Thus, 32 individuals were examined regarding the extent of their participation in the Church, which health benefits or harms they attribute to ayahuasca, what they like least and most about it, drug use schedule, psychological factors, data on childhood conduct disorder, physical examination and demographic information [59]. The results made it possible to verify that church attendance occurs once a week and that individuals are healthy [59]. Additionally, 24 of the subjects were drug or alcohol-dependent, with 22 in remission whose motivation is church attendance [59]. Similarly, 19 participants described already having a psychiatric disorder, with 8 describing that onset of remission was achieved with church participation [59].

The use of ayahuasca for therapeutic purposes has been studied mainly in terms of neurological effects, with very interesting results obtained so far [52,53,54,55,56]. However, other interesting therapeutic applications have also been proven [57,58,59], and its potential for skin healing has recently been proven [1]. The summary of the therapeutic effects is described in Table 4.

### 2.6. Effects on Metabolism

Ayahuasca has been studied in terms of its metabolism and its potential influence on it. Mello et al. [60] evaluated liver biochemical parameters in order to verify whether ayahuasca consumption influences them. For this purpose, serum was collected from 22 volunteers who had been regular consumers of ayahuasca for at least one year [60]. The results revealed that there were no significant changes in lactate dehydrogenase, alkaline phosphatase, alanine aminotransferase, aspartate aminotransferase, creatinine, urea, bilirubin and gamma glutamyl transferase, indicating that seemingly ayahuasca consumption does not affect liver function [60]. On the other hand, Madrid-Gambin et al. [61] developed a study whose objective was to evaluate the human metabolomic signature, its connection with the subjective effects and plasma concentrations of alkaloids after the consumption of ayahuasca. Thus, plasma samples were collected from 23 individuals before and after consumption of the substance [61]. Subsequently, a metabolomics analysis, an assessment of the subjective experience with Ayahuasca using the 5-Dimensional Altered States of Consciousness Rating Scale, and an integrated network analysis to determine the alkaloids present in the plasma were performed [61]. After analyzing the results, it was possible to verify that the consumption of ayahuasca altered several large neutral amino acids, decreased 2-acyl-glycerol endocannabinoids and increased N-acyl-ethanolamine endocannabinoids [61]. There was deregulation in several pathways involved in neurotransmission (synthesis of serotonin and dopamine), and some endocannabinoids and hexosylceramides were directly associated with ayahuasca alkaloids [61]. It was also possible to verify that the majority of large neutral amino acids were inversely associated with the nine 5-Dimensional Altered States of Consciousness Rating Scale. Thus, the authors concluded that subjective effects may be associated with large neutral amino acids, helping to understand the metabolic fingerprint and mechanism of action associated with ayahuasca consumption [61]. On the other hand, alkaloid concentrations do not seem to be related to these subjective effects or metabolism [61]. Riba et al. [62] evaluated the brain bioavailability of ayahuasca, as well as the time course of its effects. For this purpose, topographic quantitative electroencephalography was used in 18 volunteers after ingesting doses of ayahuasca equivalent to 0.6 and 0.85 mg of DMT/kg of body weight [62]. It was possible to verify that there was a decrease in power in the frequency bands, mainly in the theta band [62]. It was further observed that total centroid activity increased [62]. These data support 5-HT2 and dopamine receptor agonism in the effects developed on the central nervous system by ayahuasca [62]. The same authors developed a double-blind, placebo-controlled clinical trial, where they studied the pharmacokinetic profile of ayahuasca, as well as its effects on the cardiovascular level and on the urinary excretion of monoamine metabolites [18]. Thus, 18 volunteers orally received a placebo or ayahuasca containing 0.6 and 0.85 mg of DMT/kg of body weight [18]. The results showed that the higher dose caused a significant increase in diastolic blood pressure, while heart rate and systolic blood pressure increased but not significantly [18]. An increase in the urinary excretion of normetanephrine was also seen, but the levels of deaminated monoamine metabolites did not decrease [18]. These results, associated with reduced plasma levels of harmine, suggest that this compound has a predominantly peripheral metabolism (gastrointestinal and hepatic) [18]. Pharmacological effects were also evaluated by Schenberg et al. [63], who developed a study that aimed to understand the pharmacological mechanisms of action. Additionally, the correlations at the neuronal level of the modified states of consciousness associated with the consumption of ayahuasca were also studied. Thus, the compounds of ayahuasca and its metabolites in the systemic circulation were quantified, as well as an electroencephalogram recording in 20 individuals with experience in the consumption of ayahuasca after its consumption [63]. It was found that initially, there was a reduction in power in the alpha band in the brain, followed by an increase in power in the slow and fast range [63]. The first reported effects were seen in the left parieto–occipital cortex; on the other hand, the slow increase in power was observed in the left fronto-temporal, right frontal and left centro–parieto–occipital cortices [63]. The rapid increase in power was seen in the left fronto-temporal, right frontal and right parieto–occipital cortices and the left centro–parieto–occipital cortices [63]. Associated with these effects are the levels of DMT, harmine, harmaline and tetrahydroharmine and some of their metabolites [63]. These results may help in the interpretation of the cognitive and emotional effects associated with ayahuasca consumption [63]. Also, Brierley et al. [64] investigated the pharmacological mechanism, as well as the acute effects of harmine, on electrically evoked dopamine efflux parameters in the nucleus accumbens during cocaine consumption. Thus, fast cyclic voltammetry was applied to brain slices of Wistar rats in order to assess dopamine efflux in the core and shell accumbens [64]. The results indicated an increase in dopamine flux in the concha accumbens after harmine administration; however, no effect on the nucleus accumbens and reuptake in the other sub-regions [64]. The MAO inhibitor (moclobemide) had no effect on dopamine efflux, and the effect of harmine was attenuated by ketanserine (5-HT(2A/2C) antagonist) [64]. The results are indicative that harmine leads to an increase in dopamine efflux by a mechanism dependent on the presynaptic 5-HT(2A) receptor, not dependent on the activity of MAO inhibitors [64]. Thus, we may be looking at a therapy that could contribute to the therapeutic efficacy of ayahuasca for cocaine dependence [64]. Finally, Santos et al. [65] evaluated the impact of ayahuasca in terms of autonomic, neuroendocrine and immunomodulatory effects, through a double-blind randomized crossover clinical trial. The study included a placebo group, a positive control (20 mg D-amphetamine) and a group that ingested ayahuasca (1.0 mg DMT/kg body weight) [65]. Taking ayahuasca triggered subjective and neurophysiological effects absent in the positive control [65]. In both the positive control and ayahuasca groups, there was an increase in pupil diameter, cortisol levels and changes in lymphocyte subpopulations [65]. However, in the group that consumed ayahuasca, there was an increase in the levels of prolactin and natural killer cells; on the contrary, the amounts of CD4 and CD3 decreased [65]. The authors concluded that ayahuasca consumption stimulated neuroendocrine and immunomodulatory function and led to the appearance of sympathomimetic effects [65].

Thus far, studies on the effects of ayahuasca on metabolism have made it possible to understand how it works in the body [18,61,63,64]. Some effects arising therefrom, namely at the autonomic, neuroendocrine and immunological level [65], and cardiovascular and excretory levels [18], have also been investigated, allowing us to understand the effects caused by the consumption of ayahuasca at this level. The summary of the effects on metabolism is described in Table 5.

### 2.7. Physiological Effects

Over the years, the most diverse effects have been attributed to the consumption of ayahuasca, namely at the neurobiological level. Andrade et al. [14] investigated the effect of ayahuasca consumption on the neurobehavior and embryonic development of zebrafish. The animals were exposed for 96 h to amounts ranging between 0 and 1000 mg/L in order to assess toxicity, and the effects on locomotion activity were tracked through the ZebraBox video system after 120 and 144 h of exposure to amounts of 0 to 20 mg/L [14]. The results obtained were similar to those carried out in mammals, revealing that the LC_50_ of ayahuasca was 236.3 mg/L and that exposure caused significant damage, namely decreased locomotor activity, loss of balance, delay in hatching, accumulation of red blood cells and edema [14]. Also, Dakic et al. [66] investigated, *in vitro*, the effect of harmine in human neural progenitor cells derived from pluripotent stem cells. The results indicated that harmine strongly inhibited a regulator of cell proliferation and brain development, the dual-specific tyrosine phosphorylation-regulated kinase [66]. The effect of harmine analogs was further tested, and a tyrosine kinase inhibitor was found to induce the proliferation of human neural progenitor cells similarly to harmine [66]. These results may help explain how harmine-induced cell proliferation *in vitro* [66]. On the other hand, Riba et al. [67] developed a study where they investigated the alterations in the spatial distribution of the brain electrical activity induced by ayahuasca. For this purpose, low-resolution electromagnetic tomography was performed on 18 volunteers after the administration of ayahuasca (0.85 mg of DMT/kg of body weight) or a placebo [67]. Subjective effects were measured using the Hallucinogen Rating Scale [67]. The results indicated significant differences in low-resolution electromagnetic tomography between the ayahuasca group and the placebo group [67]. Additionally, there were increases in all six Hallucinogen Rating Scales [67]. It was also possible to verify that there was a decrease in power density in the theta, alpha-2, delta and beta-1 frequency bands, the latter three being found predominantly in the temporo-parieto-occipital junction, and the first in the temporomedial and lateral cortex in the frontomedial regions [67]. The authors were able to conclude that the psychological effects caused by ayahuasca are due to the involvement of the unimodal and heteromodal association cortex and the limbic structures [67]. The same authors also investigated the effects of ayahuasca on regional cerebral blood flow [20]. To this end, they performed a double-blind, randomized clinical trial, where 15 volunteers received a dose of ayahuasca equivalent to 1.0 mg DMT/kg of body weight or placebo [20]. At 100–110 min after drug administration, regional cerebral blood flow was measured using single photon emission tomography [20]. The results showed that after ayahuasca consumption, there was an increase in blood perfusion in areas implicated in subjective feeling states, somatic awareness and emotional arousal (right hemisphere of the anterior insula and anterior/frontomedial cingulate cortex) [20]. The same results were verified in the left amygdala/parahippocampal gyrus [20]. Thus, the results suggest that ayahuasca can interact with neural systems involved in interoception and emotional processing, playing a role in the serotonergic neurotransmission of these processes [20]. Finally, Viole et al. [68] evaluated the entropy of the brain’s functional connectivity by means of functional magnetic resonance imaging. Thus, the aforementioned technique was applied to the brain of human beings at rest in a normal waking state or after an alteration of consciousness due to the consumption of ayahuasca [68]. An increase in the Shannon entropy of the distribution of degrees of the networks in the ayahuasca consumer group was verified. These results are in accordance with the entropic brain hypothesis, which was also intended to be evaluated in this study [68].

Other effects at the physiological level have also been reported by several studies. Alvarenga et al. [69] evaluated the sexual performance of male Wistar rats deprived of sleep after acute consumption of ayahuasca. For this, the animals were subjected to sleep deprivation for 96 h, after which they were administered ayahuasca at 250, 500 and 1000 μg/mL [69]. A control group, where a saline solution was administered instead of ayahuasca, was also used [69]. After measuring hormone concentrations and sexual behavior, ayahuasca was found to significantly decrease sexual performance, although the lower dose increased sexual performance in sleep-deprived rats [69]. On the other hand, the 500 μg/mL dose demonstrated a detrimental effect on sexual response compared to the control group [69]. Regarding the measurement of hormone concentrations, it was found that corticosterone remained unchanged, while an increase in testosterone occurred in rats deprived of sleep with saline solution [69]. The authors were able to conclude that ayahuasca ingestion, combined with sleep deprivation, suggested a decrease in sexual performance [69]. Another study developed by Barbanoj et al. [70] aimed to investigate the influence of daytime consumption of ayahuasca on sleep parameters. Thus, 22 volunteers participated in the study and were administered an ayahuasca dose of 1 mg of DMT/kg body weight (study group), 20 mg of D-amphetamine (positive control) or a placebo (control) [70]. Subsequently, spectral analysis of sleep, polysomnography and sleep quality were evaluated [70]. The results obtained in the polysomnography allowed for verification that ayahuasca inhibits rapid eye movement sleep, increasing the onset latency as the positive control [70]. It was also verified that ayahuasca did not induce deterioration in the quality of sleep, nor interruptions in the beginning or during sleep [70]. Regarding spectral analysis, it was found that ayahuasca led to an increase in potency in the high-frequency range and potency of slow wave sleep [70]. Thus, it was possible to conclude that ayahuasca can interfere with sleep [70]. Once again, Riba et al. [71] developed a double-blind, balanced crossover project in order to evaluate the acute effects of ayahuasca in the suppression of P50 auditory evoked potential and prepulse inhibition of startle, as well as its modulatory actions in sensory and sensorimotor gating measures. The study had 18 volunteers who were given a placebo or ayahuasca in amounts of 0.6 mg and 0.85 mg DMT/kg of body weight [71]. The results showed that ayahuasca consumption did not lead to significant changes in prepulse inhibition of startle, habituation rate or startle response; however, there was a significant (dose-dependent) decrease in P50 suppression [71]. Thus, the authors could conclude that there were no effects on sensorimotor gating; however, the suppression of P50 indicated a decreasing effect of ayahuasca on sensory gating [71]. Oliveira-Lima et al. [72] investigated the effects of ayahuasca on hyperlocomotion and spontaneous locomotor activity, normally caused by ethanol consumption, as well as locomotor sensitization and counter-sensitization, in mice. After analyzing the results obtained, it was found that ayahuasca managed to prevent the development of behavioral sensitization caused by ethanol [72]. Also, in counter-sensitization, where, after sensitization, ayahuasca was administered for eight days, there was a block in the expression of sensitization [72]. Thus, ayahuasca has been shown to inhibit and reverse behaviors associated with ethanol dependence [72]. Another group investigated the acute and chronic effects of ayahuasca on the structural parameters of the aorta in rats [73]. The results were indicative that ayahuasca administration caused stretching and flattening of vascular smooth muscle cells [73]. Alterations in the arrangement and distribution of collagen and elastic fibers were also verified [73]. However, these results are still premature [73]. Serra et al. [74] also carried out a study whose objective was to evaluate the effect of ayahuasca on ethanol self-administration as well as the role of 5-HT2A receptors in these effects. For this purpose, mice with previous access to ethanol were used, which were later treated with ayahuasca or with a 5-HT2A receptor antagonist [74]. It was found that after treatment with ayahuasca, the expression of ethanol self-administration decreased, as well as ethanol intake and preference [74]. Additionally, administration of the 5-HT2A receptor antagonist was found to block the effects of ayahuasca on ethanol consumption without significantly attenuating ethanol self-administration [74]. These results support the potential of ayahuasca as therapies for the treatment of alcohol dependence [74]. Finally, Frecska et al. [75] evaluated the effect of ayahuasca on dichotic stimulus alternation. Thus, 10 volunteers, participants in ayahuasca ceremonies, performed binocular rivalry tests before and after ingestion of ayahuasca [75]. It was found that the consumption of the substance under study led to an increase in the time of dominance both in standard conditions of binocular rivalry and with binocular rivalry, with periods of dominance being longer when drinking the beverage [75]. These results may be related to slow visual processing and the increase in dominance may be the result of hallucinogen-induced alteration of gamma oscillations in the visual pathways [75]. Later, the same research group tested the alternation of perception in ayahuasca ceremonial participants, in order to examine whether alteration of interhemispheric function occurs under the influence of hallucinogens [76]. The results indicated an increase in the length of a percept, but a decrease in rivalry alternation rates [76]. Thus, the results are in agreement with previous studies that support the occurrence of interhemispheric fusion in altered states of consciousness [76].

The most studied physiological effects are, once again, those associated with neurobiology [14,20,66,67,68]. However, it is worth highlighting some interesting studies that looked into different but equally important parameters, such as the influence of ayahuasca on sleep parameters [70] or on sexual performance [69]. The summary of the physiological effects is described in Table 6.

### 2.8. Toxicological Effects and Toxicity

The plants used in traditional medicine are taken as safe; however, some can be toxic, especially depending on doses, making it crucial to investigate their possible toxicity [77]. Kummrow et al. [77] developed a study where they evaluated the mutagenicity of two ayahuasca samples and two individual drinks from *P. viridis* and *B. caapi* plants, using the Salmonella/microsome assay with TA98 and TA100. Additionally, harmine and harmaline have also been tested [77]. The results obtained indicated that both ayahuasca drinks were mutagenic for TA98 and TA100. Also, harmaline and *B. caapi* drink showed mutagenicity for TA98. On the other hand, the *P. viridis* drink and harmine were not mutagenic [77]. In another study developed by Colaço et al. [78], the potentially toxic effects of ayahuasca were evaluated *in vivo*. For this purpose, rats were chronically exposed to the drink, and the levels of monoamines (serotonin, dopamine and norepinephrine), their metabolites and the brain-derived neurotrophic factor in the brain were evaluated [78]. Thus, female and male Wistar rats were used, divided into five groups: control group (water administration), fluoxetine group (administration of fluoxetine (selective serotonin reuptake inhibitor antidepressant)), Aya0.5 group (administration of ayahuasca 0.5× the ritualistic dose), Aya1 group (administration of ayahuasca 1× the ritualistic dose) and Aya2 group (administration of ayahuasca 2× the ritualistic dose). The results showed that ayahuasca was safe for the rats, with serotonin levels significantly increased in the Aya2 group and its 5-HIAA metabolite significantly decreased in the fluoxetine group [78]. Regarding dopamine and the HVA metabolite, no significant changes were observed between experimental groups; however, the DOPAC metabolite increased significantly in the Aya1 and Aya2 groups, the DOPAC/dopamine turnover was significantly higher in the Aya2 group, and the HVA/DOPAC ratio was significantly lower in the male groups Aya0.5, Aya1 and Aya2 [78]. Regarding norepinephrine, it was not detected, and its metabolite did not change significantly [78]. With regard to brain-derived neurotrophic factors in the hippocampus, there was a significant increase in the FLX and Aya2 female groups [78]. Also, Pic-Taylor et al. [79] carried out a study in female Wistar rats to assess the acute toxicity of ayahuasca, following the OECD Guide 423/2001 protocol. Thus, ayahuasca doses of 30× and 50× that of the dose taken during a religious ritual were administered by gavage, and the animals were observed for 14 days [79]. The behavior of the animals was evaluated by carrying out open field, elevated plus maze and forced swimming tests. After analyzing the results, it was possible to verify that the groups treated with ayahuasca significantly decreased locomotion in the open field test and elevated plus maze tests compared to the control [79]. Regarding the forced swimming test, the rats treated with ayahuasca swam more. Additionally, this group also showed greater neuronal activation in all brain areas involved in serotonergic neurotransmission [79]. No permanent damage has been detected, although there may be some brain damage [79]. In another study developed by Motta et al. [80], Wistar rats were also used in order to assess the development of maternal and fetal toxicity. The rats were divided into five groups: a control and four groups with four different doses of ayahuasca (1×, 2×, 4× and 8× the typical dose taken by an adult during an ayahuasca ritual). After analyzing the results, it was possible to verify that the control group and the two groups with the lowest dose of ayahuasca survived; however, the groups where ayahuasca was administered 4× and 8× only survived 56% and 48%, respectively, presenting kidney damage [80]. Additionally, the group receiving the 8× dose showed neuronal losses in the hippocampus and raphe nuclei, intrauterine growth retardation, induced embryonic deaths, and increased occurrence of fetal anomalies [80]. The group whose administered dose was 2× showed a neuronal loss in the CA1 zone of the hippocampus [80]. At non-lethal doses, ayahuasca increased embryonic death and the incidence of fetal anomalies (soft tissue and skeleton) [80]. These results suggest that ayahuasca may have developmental toxicity and that its use by pregnant women may pose risks to the fetus [80]. Finally, Simão et al. [81] carried out a study with the objective of studying the cytotoxic effects of five preparations of ayahuasca infusions in dopaminergic immortalized cell lines. After evaluating the cytotoxicity, the results suggested that a concentration of 10 µM of harmine and THH, induce cytotoxicity in the N27 cell line [81]. The compound harmaline also induced cytotoxicity at a concentration of 80 µM, contrary to DMT, which did not showed cytotoxicity in the range of concentrations tested (0.0008 to 1 µM) [81]. Regarding the decoction extracts, it was found that *P. harmala* showed cytotoxicity at concentrations corresponding to 16 and 80 µM of harmaline, *B. caapi* showed cytotoxicity at concentrations corresponding to 10 µM of harmine and a commercial mixture showed cytotoxicity at the corresponding concentrations of 10 µM THH [81]. The total protein was also quantified and the values agreed with those obtained in the cytotoxicity assays (when cell viability decreased, the same was verified in the total protein contents) [81]. Some of these studies indicated that ayahuasca consumption is safe [78], not leading to permanent damage [79]. However, alterations or lack of knowledge about the doses usually consumed could have serious consequences, namely at the level of dopaminergic neurons [81]. The results obtained in the study developed by Motta et al. [80], where several negative consequences associated with the consumption of ayahuasca during pregnancy were verified, since, although not advised, the decision to consume will be made by the woman herself. The summary of the toxicological effects and toxicity is described in Table 7.

Given the presented studies, it was possible to verify that for some decades, interest in the therapeutic effects associated with ayahuasca has aroused much interest. It is clear that most studies focus on psychological/psychiatric disorders, and, in fact, many results point to the use of ayahuasca as a possible treatment, being depression and anxiety the most studied topics. However, several studies were carried out on ayahuasca rituals or those that resort to individuals who are regular consumers of ayahuasca and who have religious beliefs in this beverage. Thus, these results should be considered with caution, as they may sometimes present this bias. Despite this, many other studies are clinical trials controlled by a placebo and, therefore, present more reliable results. However, despite the positive results, the mechanism by which ayahuasca acts is still not completely understood, from a molecular point of view, nor which are its most promising components to be used as future anxiolytics or antidepressants. The same can be seen in the treatment of addictions using ayahuasca. It must be considered that ayahuasca, despite all the beneficial effects associated, is a psychoactive substance, so we may be compensating for one addiction with another. The same can be considered for other reported therapeutic effects.

Contrary to what usually happens with other substances, most of the studies dealing with ayahuasca are clinical trials or used by volunteers. However, it would be important to understand and complete the findings of these studies with other primary studies, namely *in vitro* and *in vivo* assays. Although it is undeniable that, in fact, ayahuasca presents some benefit, it would be important to study and understand what makes this possible.

## 3. Materials and Methods

### 3.1. Data Acquisition

We intend to identify the works published until December 2022, when the biological or bioactive properties/effects associated with ayahuasca were evaluated.

### 3.2. Search Strategy

Electronic searches were performed in PubMed (up to December 2022). The following keywords were used: “ayahuasca AND biological effects”; “ayahuasca AND biological activity”; “ayahuasca AND bioactive effects” and “ayahuasca AND bioactive activity”.

### 3.3. Eligibility Criteria

For the purposes of this review, *in vitro*, *in vivo*, clinical trials or studies that used ayahuasca extracts to study biological and bioactive properties with therapeutic purposes were included. Studies that used the ayahuasca beverage in its original form, or prepared with other analogs, were considered. Additionally, studies that used plant extracts used in the preparation of ayahuasca (original or altered) were also included. Exclusion criteria consisted of not fitting the theme, review articles, interviews, opinion pieces or comments, letters or editorials, conference abstracts or posters, case reports and published abstracts. Only articles written in English were included.

### 3.4. Study Selection and Data Extraction

Following the PRISMA (Preferred Reporting Items for Systematic Reviews and Meta-Analyses) recommendations [82,83,84], two authors independently screened all titles and abstracts based on the defined inclusion criteria. Subsequently, the full text of each potentially eligible article was obtained and screened to support its inclusion in this systematic review. Any disagreement about study eligibility was solved through discussion between authors.

According to the PRISMA methodology [82,83,84], two authors independently reviewed and extracted the data using a prespecified protocol. In cases of discordance, a third author was consulted to analyze discrepancies in data extraction.

## 4. Conclusions

Treatments based on products of natural origin have gained prominence over the years. Despite the wide variety of drugs currently available, some trigger serious adverse effects, which can limit the daily lives of users. Additionally, despite global development, there are still many people in need and without access to basic health care, finding in plant specimens the only available cure.

In general, the available studies indicate that the therapeutic use of ayahuasca can be effective and bring benefits in some conditions, the most evident in terms of psychological disorders. In fact, studies that evaluate the therapeutic effects of ayahuasca in terms of emotional well-being and mental health are the most common and with the most evident results. However, the treatment of substance imbalances and addictions has also had some focus with regard to ayahuasca consumption. The interest and demand for favorable results associated with the consumption of ayahuasca has also been growing, as evidenced by the increase in publications addressing the topic. Other approaches, particularly at the level of molecular biology and microbiology, have also shown that ayahuasca may have promising effects in combating pathologies associated with microorganisms and in the treatment of dermal lesions or neurodegenerative diseases. It is notorious that most of the available studies were performed on volunteers in rituals or clinical trials, which is surprising when compared with the small number of existing *in vivo* and *in vitro* reports. However, it should also be noted that many of the existing articles are observational studies, and many were carried out on volunteers who participate in religious rituals. Thus, we have to consider that some of the results may present a bias justified by the spiritual and religious beliefs of the volunteers, constituting an important limitation of the results presented. Additionally, more studies at the molecular level are needed to understand the possible therapeutic effects of this substance better.

## Figures and Tables

**Table 1:** Summary of the results obtained for effects associated with mental health and psychological well-being.

Studied Sample	Main Results	Reference
Ayahuasca beverage	Reduction in anxiety and depression behaviors	[27]
	Increased anxiety (childhood), memory impairment (adolescence), unchecked changes (adult)	[28]
	Decreased depression, increased psychoactivity, increased blood perfusion in regions that regulate emotions and mood	[29]
	No change in anxiety, decrease in panic and hopelessness	[30]
	Mindfulness reduced the severity of depression and depressive symptoms	[31]
	No decrease in ethanol intake. Ethanol increased cFos expression after being treated with ayahuasca	[32]
	The effectiveness of ayahuasca in treating addiction depends on guidance during the ritual	[33]
	Potential substance addiction treatment and relapse prevention	[34]
	Improved impulsivity, compassion, attachment and spiritual acceptance, self-transcendence and novelty seeking.	[35]
	Benefits in some psychological and interpersonal dimensions, biographical memories, emotional release and contact experiences with the deceased	[36]
	Improvements in affect and thinking style, life satisfaction and mindfulness. Decreased depression and stress. Correlation between changes in affect, life satisfaction and mindfulness and the level of ego dissolution	[37]
	placebo-related beneficial mental health effects	[38]
	Decreased internal reactivity and judgmental processing of experiences. Increased decentralization capacity	[39]
	Increased healthier diets, reduced alcohol intake, improved mood, self-acceptance and relationships	[40]
	Improved tranquillity, visual phenomena, insights, numinosity. Decreased psychiatric symptoms. Increased serenity, vivacity/cheerfulness and assertiveness	[41]
	Improved psychological effects. Tolerability at the cardiovascular level.	[42]
	Improvement in emotional non-acceptance, emotional interference, lack of control, state of consciousness and decentralization. No change in mindfulness abilities.	[43]
	Decrease in the values of achievement in life, living valued, self in close relationships, self in social relationships and general self. Improved results for positive self and decentralization	[44]
	Enhancement of divergent creative thinking and increased psychological flexibility	[26]
	Increased phosphenic and original responses, visual creativity and entoptic activity	[45]
	Alteration of neuroticism and moderation of personality	[46]
	Decreased temporal distortion	[47]
	Increased Peak Experience Profile and Spiritual Well-being at Mysticism	[48]

**Table 2:** Summary of the results obtained for effects associated with antimicrobial properties.

Studied Sample	Main Results	Reference
141 plant species	Inhibition of growth of *E. coli* and *S. aureus*	[49]
*P. viridis*, *B. caapi*, *M. hostilis*, *P. harmala* and a commercial mixture of beverages	Inhibition of bacterial growth in all strains. Anti-quorum sensing properties. Anti-biofilm activity in two samples on the same strain.	[7]

**Table 3:** Summary of the results obtained for effects associated with anti-inflammatory properties.

Studied Sample	Main Results	Reference
*P. viridis*, *B. caapi*, *M. hostilis*, *P. harmala* and a commercial mixture beverages	Presentation of anti-inflammatory activity	[7]
Ayahuasca beverage	Negative correlation between C-reactive protein and serum cortisol levels. Decreased levels of C-reactive protein and correlation between greater reductions in C-reactive protein and less depressive symptoms	[51]
*P. harmala* beverage	Harmine showed anti-inflammatory effects by inhibiting the NF-kB signaling pathway	[50]

**Table 4:** Summary of the results obtained for effects associated with other therapeutics effects.

Studied Sample	Main Results	Reference
*P. viridis*, *B. caapi* extracts and Harmine and DMT	Neuroprotective effect on crude extracts and hydroalcoholic fractions. Stimulation of cell proliferation and neuroprotection profile at lower doses	[52]
DMT	Increased proliferation of neural stem cells, migration of neuroblasts, promoting the generation of new neurons. Increased adult neurogenesis and improved learning tasks and spatial memory.	[53]
*B. caapi* extracts	Therapeutic potential of *B. caapi* stem extract in the treatment of Parkinsonism and other neurodegenerative disorders	[54]
*B. caapi* extract, harmine and harmaline	Therapeutic potential of *B. caapi* extract in Parkinson’s disease	[55]
*P. viridis*, *B. caapi*, *M. hostilis*, *P. harmala* and a commercial mixture beverages	Potential wound-healing effect	[1]
Ayahuasca beverage	Decreased working memory. Decreased disability by promoting neuromodulatory or compensatory effects on executive function	[56]
	Reductions in thoughts and symptoms related to eating disorders, anxiety, depression, self-harm, suicide and problematic substance use	[57]
	Significant decrease in growth hormone, heart rate and systolic blood pressure. No differences in autonomic, neurophysiological or immunological effects	[58]
	Remission of individuals dependent on substances or with a psychiatric disorder after participation in the church	[59]

**Table 5:** Summary of the results obtained for effects associated with effects on metabolism.

Studied Sample	Main Results	Reference
Ayahuasca beverage	No changes in liver function	[60]
	Changes in several large neutral amino acids, decrease in 2-acyl-glycerol endocannabinoids and increase in N-acyl-ethanolamine endocannabinoids. Dysregulation of various neurotransmission pathways. Subjective effects associated with large neutral amino acids. Alkaloid concentrations unrelated to subjective effects or metabolism	[61]
	Decreased power in frequency bands. Increased total centroid activity. Support of 5-HT2 and dopamine receptor agonism in ayahuasca-induced effects	[62]
	Increased diastolic blood pressure, heart rate and systolic blood pressure. Increased urinary excretion of normetanephrine without decrease in deaminated monoamine metabolite levels. Results indicative of a predominantly peripheral metabolism of harmine	[18]
	Decreased alpha-band potency in the brain and increased slow- and fast-range potency associated with levels of DMT, harmine, harmaline and tetrahydroharmine and some of their metabolites	[63]
	Stimulation of neuroendocrine and immunomodulatory function and led to the appearance of sympathomimetic effects	[65]
Harmine	Increased dopamine flux in the concha accumbens after harmine administration. Harmine leads to an increase in dopamine efflux by a presynaptic 5-HT(2A) receptor-dependent mechanism. Possible therapy for cocaine addiction	[64]

**Table 6:** Summary of the results obtained for effects associated with physiological effects.

Studied Sample	Main Results	Reference
Harmine	Inhibition of the regulator of cell proliferation and brain development by harmine. Induction of proliferation of human neural progenitor cells by a harmine analog	[66]
Ayahuasca beverage	Decreased locomotor activity, loss of balance, delayed hatching, accumulation of red blood cells and edema	[14]
	Significant differences in low-resolution electromagnetic tomography between groups. Decreased power density in the theta, alpha-2, delta and beta-1 frequency bands. Possible involvement of the unimodal and heteromodal association cortex and limbic structures in the psychological effects caused by ayahuasca.	[67]
	Increased blood perfusion in areas implicated in subjective feeling states, somatic awareness, emotional arousal and left amygdala/parahippocampal gyrus. Possible association of ayahuasca with the neurotransmission of neural systems involved in interoception and emotional processing.	[20]
	Increase in the Shannon entropy of the degree distribution of networks	[68]
	Ayahuasca ingestion, combined with sleep deprivation, decreased sexual performance	[69]
	Inhibition of rapid eye movement sleep by increasing onset latency. No induction of deterioration in sleep quality nor interruption of sleep. Increased power in the high-frequency range and slow-wave sleep power	[70]
	No significant changes in prepulse inhibition of startle, habituation rate or startle response. Significant decrease in P50 suppression. No effects on sensorimotor gating, but decreasing effect on sensory gating	[71]
	Prevention of the development of behavioral sensitization caused by ethanol. Block in the expression of sensitization. Inhibition and reversal of behaviors associated with ethanol dependence.	[72]
	Stretching and flattening of vascular smooth muscle cells. Changes in the arrangement and distribution of collagen and elastic fibers	[73]
	Potential therapeutic effect of ayahuasca in the treatment of alcohol dependence	[74]
	Increased dominance time under standard binocular rivalry conditions such as binocular rivalry	[75]
	Increased percept length, and decreased rivalry switching rates	[76]

**Table 7:** Summary of the results obtained for effects associated with toxicological effects and toxicity.

Studied Sample	Main Results	Reference
*P. viridis* and *B. caapi* beverages; Harmine and harmaline	Mutagenicity induced by ayahuasca beverages for TA98 TA100. Mutagenicity induced by harmaline and *B. caapi* drink for TA98. Drink of *P. viridis* and harmine without mutagenicity	[77]
Ayahuasca beverage	Increased serotonin (Aya2) levels and decreased 5-HIAA metabolite. No changes for dopamine and HVA metabolite, but increased DOPAC metabolite (Aya1 and Aya2), increased DOPAC/dopamine turnover (Aya2) and decreased HVA/DOPAC ratio in male groups (Aya0.5, Aya1 and Aya2). Increased brain-derived neurotrophic factor in female groups (FLX and Aya2).	[78]
	Decreased locomotion in the open field test elevated plus maze tests. Increased movement in the forced swimming test and increased neuronal activation in all brain areas involved in serotonergic neurotransmission. No permanent damage	[79]
	56% and 48% survival for 4× and 8× ayahuasca with kidney damage. Neuronal losses in the hippocampus and raphe nuclei, intrauterine growth retardation, induced embryonic deaths and increased occurrence of fetal anomalies	[80]
*P. viridis*, *B. caapi*, *M. hostilis*, *P. harmala* and a commercial mixture beverages	Induction of cytotoxicity in the N27 cell line by harmine, harmaline and THH and by extracts of *P. harmala*, *B. caapi* and a commercial mixture. No cytotoxicity for DMT.	[81]

## Supplementary Materials

The following supporting information can be downloaded at: https://www.mdpi.com/article/10.3390/plants12132573/s1, Table S1: Main characteristics of the included studies in this systematic review.
