# Supplementary material for: A Systematic Review on the Therapeutic Effects of Ayahuasca
Source: Plants (Basel). 2023 Jul 7;12(13):2573. doi: 10.3390/plants12132573 (PMC10346174; doi:10.3390/plants12132573)
Supplement: Supplementary file 1 [file plants-12-02573-s001.zip › plants-2444359-supplementary.pdf]

Supplementary Table S1. Main characteristics of the included studies in this systematic review.

Reference	Authors	Year	Type of study	Study object (sample size (if applicable))	Studied compound/extract	Methodology	Evaluated parameters
1	Gonçalves *et al.*	2022	*In vitro*	NHDF cells	*P. viridis*, *B. caapi*, *M. hostilis*, *P. harmala* and a commercial mixture beverages	MTT assay; Wound-Healing assay; Parallel Artificial Membrane Permeability assay	Wound-Healing Potential
7	Gonçalves *et al.*	2020	*In vitro*	*Staphylococcus aureus*, *Bacillus* cereus, *Listeria monocytogenes*, *Enterococcus* faecalis, *Acinetobacter baumannii*, *Pseudomonas aeruginosa*, *Escherichia coli*, and *Salmonella* Typhimurium	*P. viridis*, *B. caapi*, *M. hostilis*, *P. harmala* and a commercial mixture beverages	Folin–Ciocalteu colorimetric method; aluminum chloride colorimetric method; DPPH Scavenging Assay; -Carotene Bleaching Test; protein denaturation inhibition assay; Disc Diffusion Assay; Resazurin Microtiter Method; Anti-Quorum Sensing Properties: Solid Diffusion Assay; Anti-Biofilm Activity evaluation by scanning electron microscopy	Antioxidant, Anti-Inflammatory and Antimicrobial activity
14	Andrade *et al.*	2018	*In vivo*	Zebrafish (not specified)	Ayahuasca beverage	Fish embryo toxicity test; behavioral assessment	Embryo development effects and neurobehavior
18	Riba *et al.*	2003	Clinical trial	Human volunteers (18)	Ayahuasca beverage	Visual analog scales;Hallucinogen Rating Scale; Addiction Research Center Inventory;	Subjective and cardiovascular effects and alkaloid pharmacokinetics

						Cardiovascular Measures; Pharmacokinetic Analysis	
20	Riba *et al*.	2006	Clinical trial	Human volunteers (15)	Ayahuasca beverage	Hallucinogen Rating Scale; Addiction Research Center Inventory; single photon emission tomography imaging	Regional cerebral blood Flow effects
26	Kuypers *et al*.	2016	Ayahuasca ritual	Human volunteers (26)	Ayahuasca beverage	Pattern/line meanings test; picture concept test; visual analog scales	Creative thinking effects
27	Silva *et al*.	2022	*In vivo*	Rats (80)	Ayahuasca beverage	Open field test; forced swimming test	Behavioral response in neuroinflammation
28	Correa-Netto *et al*.	2017	*In vivo*	Mice (8-12/group)	Ayahuasca beverage	Behavioral tests; Open field test; elevated plus maze tasks; Morris water maze	Memory and anxiety effects
29	Sanches *et al*.	2016	Clinical trial	Human volunteers (17)	Ayahuasca beverage	Hamilton Rating Scale for Depression, the Montgomery-Åsberg Depression Rating Scale; Brief Psychiatric Rating Scale; Young Mania Rating Scale; Clinician Administered Dissociative States Scale	Anti-depressive potentials and its effects on regional cerebral blood flow
30	Santos *et al*.	2007	Observational study	Human volunteers (9)	Ayahuasca beverage	Standard questionnaires to evaluate state-anxiety, trait-anxiety, panic-like and hopelessness	Psychometric measures of anxiety, panic-like and hopelessness effects
31	Mian *et al*.	2019	Observational study	Human volunteers (152)	Ayahuasca beverage	Behavioral Activation for Depression Scale-Short	Contribution of mindfulness and

						Form; Five Facet Mindfulness Questionnaire; Experiences Questionnaire; Center for Epidemiological Sciences Depression Short Form	behavioral activation in anti-depressant effects
32	Nolli *et al*.	2020	*In vivo*	Wistar rats (64)	Ayahuasca beverage	cFos immunohistochemistry	Treatment to decrease ethanol intake; neural activity effects
33	Talin *et al*.	2017	Observational study	Human volunteers (not specified)	Ayahuasca beverage	Long-term fieldwork and participant observation in ayahuasca communities	Interactive ritual contexts support on the healing effort
34	Loizaga-Velder *et al*.	2014	Observational study	Human volunteers (not specified)	Ayahuasca beverage	Review of therapeutic projects; interviews with therapists who apply ayahuasca in the treatment of addictions; interviews with expert researchers on the topic; interviews with individuals who had undergone ayahuasca-assisted therapy for addiction in diverse treatment settings	Substance dependence treatment
35	Peláez *et al*.	2020	Observational study	Human volunteers (14)	Ayahuasca beverage	Scale Temperament and Character Inventory–Revisited; Cloninger's model	Personality traits
36	González *et al*.	2019	Observational	Human	Ayahuasca	General Characteristics	Grief therapy

			study	volunteers (60)	beverage	Bereavement Questionnaire; Texas Revised Inventory of Grief; Acceptance and Action Questionnaire; Ethical Considerations	
37	Uthaug *et al.*	2018	Ayahuasca ritual	Human volunteers (57)	Ayahuasca beverage	Picture concept task; Depression, Anxiety, and Stress Scale-21; Satisfaction with Life Scale; Five Facets Mindfulness Questionnaire; Ego Dissolution Inventory	Well-being and cognitive thinking style effects; Depend on the degree of ego dissolution effects
38	Uthaug *et al.*	2021	Clinical trial	Human volunteers (30)	Ayahuasca beverage	Multifaceted empathy test; Ego Dissolution Inventory; the 5-Dimensional Altered States of Consciousness Rating Scale; Depression, Anxiety, and Stress Scale 21; Brief Symptom Inventory 18; Five Facets Mindfulness Questionnaire	Mental health changes
39	Soler *et al.*	2016	Observational study	Human volunteers (25)	Ayahuasca beverage	Five Facets Mindfulness Questionnaire; Experiences Questionnaire	Psychological mechanisms underlying the beneficial effects
40	Harris *et al.*	2012	Observational study	Human volunteers (177)	Ayahuasca beverage	Qualitative questionnaire	Effects on joy in life, relationship to the sacred and toxic feelings
41	Barbosa *et al.*	2005	Observational study	Human volunteers (28)	Ayahuasca beverage	Clinical Interview Schedule-Revised Edition;	Psychological aspects in mental

						Sociodemographic profile; Inventory of intrinsic religious beliefs profiles; Inventory of expectancies/motivations; Phenomenological mapping of the altered states of Consciousness; Behavioral changes inventory	health
42	Riba *et al*.	2001	Clinical trial	Human volunteers (6)	Ayahuasca beverage	Visual analogue scales; Spanish adaptations of the Hallucinogen Rating Scale; Addiction Research Center Inventory; Tolerability measures	Psychological effects and tolerability
43	Domínguez-Clavé *et al*.	2019	Observational study	Human volunteers (45)	Ayahuasca beverage	Difficulties in Emotion Regulation Scale; mindfulness traits (Five Facet Mindfulness Questionnaire–Short Form; Experiences Questionnaire	Emotion regulation and mindfulness-related abilities effects
44	Franquesa *et al*.	2018	Observational study	Human volunteers (122)	Ayahuasca beverage	Brief Symptom Inventory 18 scale; Psychoticism scale of the Symptoms Assessment-45; Experiences Questionnaire; Engaged Living Scale; Experiencing of Self Scale	Link between Decentering, Values and Self
45	Frecska *et al*.	2012	Ayahuasca ritual	Human volunteers (61)	Ayahuasca beverage	Torrance Tests of Creative Thinking	Creativity and expression of

							creativity
46	Weiss *et al*.	2021	Ayahuasca ritual	Human volunteers (256)	Ayahuasca beverage	Changes in personality traits by the Five-Factor model; demographic characteristics, baseline personality, and acute post-ayahuasca experiences	Personality changes
47	Campagnoli *et al*.	2020	Ayahuasca ritual	Human volunteers (9)	Ayahuasca beverage	Hearing and reproduction of musical stimuli	Effects of listening to musical stimuli on subjective time
48	Trichter *et al*.	2009	Observational study	Human volunteers (54)	Ayahuasca beverage	Peak Experience Profile; Spiritual Well-being Scale; Mysticism Scale	Influence on spirituality
49	Bussmann *et al*.	2010	*In vitro*	*Escherichia coli* and *Staphylococcus aureus*	141 plant species	Minimal inhibitory concentration	Minimum inhibitory concentration and antibacterial properties determination
50	Liu *et al*.	2017	*In vitro* and *in vivo*	HEK-293T cells and mice (not specified)	*P. harmala* beverage	Gene reporter assay; Determination of nitric oxide production; Enzyme-linked immunosorbent assay; Quantitative real-time PCR; Immunofluorescence staining and microscopic imaging; H&E staining	Anti-inflamató Anti-inflammatory effects
51	Galvão-Coelho *et al*.	2020	Clinical trial	Human volunteers (73)	Ayahuasca beverage	Blood inflammatory biomarkers measure; Montgomery-Åsberg	Blood inflammatory biomarkers

						Depression Rating Scale	
52	Katchborian-Neto *et al.*	2020	*In vitro*	SH-SY5Y neuroblastoma cells	*P. viridis*, *B. caapi* extracts and Harmine and DMT	MTT assay; Ki-67 staining (cell proliferation); calcein-AM/PI staining	Neuroprotector potential
53	Morales-Garcia *et al.*	2020	*In vitro* and *in vivo*	neural stem cells and C57/BL6 mice (24)	DMT	Growth and proliferation measurements; differentiation of cultures; western blot analysis; immunocytochemistry; immunohistochemistry; cell count analysis; behavioral studies	Neurogenic effects
54	Samoylenko *et al.*	2010	*In vitro*	Recombinant human brain monoamine oxidase -A and -B	*B. caapi* extracts	Inhibition kinetics assay; cytotoxicity assay; Determination of ROS assay; Ensaio de inibição da atividade das enzimas acetilcolinesterase, butirilcolinesterase e catecol-O-metil transferase	Prevention of neurological disorders through the antioxidant and inhibitory activity of Monoamine Oxidase
55	Schwarz *et al.*	2003	*In vitro*	Wistar rats liver	*B. caapi* extract, harmine and harmaline	MAO inhibition assay; *In vitro* release of [3H]dopamine ([3H]DA)	Investigation of *in vitro* activity relevant to Parkinson's Disease
56	Bouso *et al.*	2013	Observational study	Human volunteers (24)	Ayahuasca beverage	Tower of London task; Stroop color and word test; Sternberg working memory task	Neuropsychological performance (working memory and executive function) effects
57	Lafrance *et al.*	2017	Observational	Human	Ayahuasca	Interview with questions of	Potential

			study	volunteers (16)	beverage	etiological, clinical, and treatment histories	therapeutic in eating disorders
58	Santos *et al*.	2012	Clinical trial	Human volunteers (17)	Ayahuasca beverage	visual analog scales; Hallucinogen Rating Scale; Addiction Research Center Inventory; Neurophysiological measures; Cardiovascular measures; Autonomic measures; Neuroendocrine measures	Tolerance or sensitization effects
59	Halpern *et al*.	2008	Observational study	Human volunteers (32)	Ayahuasca beverage	Structured Clinical Interview for DSM-IV Disorders; 14-item Hamilton Anxiety Rating Scale; 21-item Hamilton Depression Rating Scale; Symptom Check List 90 Revised; Uplifts, Hassles, Stresses, and Cognitive Failures questionnaire; Wender Utah Rating Scale for attention-deficit hyperactivity Disorder; Structured Clinical Interview for DSM-IV Axis II Personality Disorders	Beneficial effects
60	Mello *et al*.	2018	Clinical trial	Human volunteers (22)	Ayahuasca beverage	Evaluation of biochemical parameters	Hepatic biochemical parameters
61	Madrid-Gambin *et al*.	2022	Clinical trial	Human volunteers (23)	Ayahuasca beverage	5-Dimension Altered States of Consciousness Rating	Human metabolomics

						Scale	signature investigation
62	Riba *et al*.	2002	Clinical trial	Human volunteers (18)	Ayahuasca beverage	Topographic quantitative-electroencephalography; Hallucinogen Rating Scale	Cerebral bioavailability and time-course
63	Schenberg *et al*.	2015	Clinical trial	Human volunteers (30)	Ayahuasca beverage	Hallucinogen Rating Scale-Brazilian Version; Electroencephalography	Effects of oscillatory activity of different brain regions
64	Brierley *et al*.	2013	*In vitro*	Rat brain slices	Harmine	Fast cyclic voltammetry; Carbon fibre microelectrodes	Acute effects and pharmacological mechanism on electrically evoked dopamine efflux parameters
65	Santos *et al*.	2011	Clinical trial	Human volunteers (10)	Ayahuasca beverage	Hallucinogen Rating Scale; Addiction Research Center Inventory; autonomic measures; neuroendocrine measures; lymphocyte subpopulations measures; electroencephalographic measures	Autonomic, neuroendocrine, and immunomodulatory effects
66	Dakic *et al*.	2016	*In vitro*	Human embryonic stem cells	Harmine	Cell proliferation (Immunocytochemistry); cell death; DNA damage	proliferation of human neural progenitor effects
67	Riba *et al*.	2004	Clinical trial	Human volunteers (18)	Ayahuasca beverage	Hallucinogen Rating Scale; self-report questionnaire measuring psychedelicinduced subjective effects;	Changes in brain electrical activity

						topographic pharmaco-electroencephalography; low-resolution electromagnetic tomography	
68	Viol *et al*.	2017	Clinical trial	Human volunteers (10)	Ayahuasca beverage	Complex networks measures	Differences in complex networks with and without ayahuasca influence
69	Alvarenga *et al*.	2014	*In vivo*	Rats (80)	Ayahuasca beverage	Sexual behavior evaluation; paradoxical sleep deprivation; hormone concentrations evaluation	Sexual performance (sleep deprived)
70	Barbanoj *et al*.	2008	Clinical trial	Human volunteers (22)	Ayahuasca beverage	Subjective sleep quality; polysomnography; spectral analysis	Sleep parameters effects
71	Riba *et al*.	2002	Clinical trial	Human volunteers (18)	Ayahuasca beverage	P50 elicitation and recording; Startle reflex elicitation and recording; Hallucinogen Rating Scale; Spanish version of the Altered States of Consciousness Questionnaire	P50 suppression and PPI effects
72	Oliveira-Lima *et al*.	2015	*In vivo*	Mice (146)	Ayahuasca beverage	Open-field test	Ethanol treatment
73	Pitol *et al*.	2015	*In vivo*	Rats (40)	Ayahuasca beverage	Morphometric Analysis (qualitative and quantitative)	Structural parameters in aorta effects
74	Serra *et al*.	2022	*In vivo*	Mice (90)	Ayahuasca beverage	behavioral observation	Role of 5-HT2A receptors in the

							treatment of ethanol withdrawal
75	Frecska *et al*.	2004	Clinical trial	Human volunteers (10)	Ayahuasca beverage	Binocular rivalry test	Influence of binocular rivalry on high rates of dichotic stimulus alternation investigation
76	Frecska *et al*.	2003	Ayahuasca ritual	Human volunteers (10)	Ayahuasca beverage	Binocular rivalry test	Influence of binocular rivalry in revealing the temporal characteristics of brain dominance in an altered state of consciousness investigation
77	Kummrow *et al*.	2019	*In vitro*	TA98 and TA100 strains of *Salmonella enterica* serovar Typhimurium	*P. viridis* and *B. caapi* beverages; Harmine and harmaline	Salmonella/MicrosomeAssay	Mutagenicity
78	Colaço *et al*.	2020	*In vivo*	Wistar rats (85)	Ayahuasca beverage	Open field test; elevated-plus-maze apparatus test; hematological and biochemical evaluation	Toxic effects
79	Pic-Taylor *et al*.	2015	*In vivo*	Wistar rats (6/condition)	Ayahuasca beverage (*P. viridis* and *B. caapi*)	Open field, elevated plus maze, and forced swimming tests; neuronal activation (c-fos marked neurons); toxicity (Fluoro-	Behavioural and neurotoxic effects

						Jade B and Nissl/Cresyl staining)	
80	Motta *et al*.	2018	*In vivo*	Rats (130)	Ayahuasca beverage	Histological evaluation	Maternal and developmental toxicity
81	Simão *et al*.	2020	*In vitro*	Dopaminergic cells	*P. viridis*, *B. caapi*, *M. hostilis*, *P. harmala* and a commercial mixture beverages	Cellular viability assay; protein quantification;	Cytotoxicity
